# Two TRPV1 receptor antagonists are effective in two different experimental models of migraine

**DOI:** 10.1186/s10194-015-0539-z

**Published:** 2015-06-24

**Authors:** Jannis E Meents, Jan Hoffmann, Sandra R Chaplan, Lars Neeb, Sigrid Schuh-Hofer, Alan Wickenden, Uwe Reuter

**Affiliations:** Department of Physiology, Uniklinik RWTH Aachen, Pauwelsstr. 30, D-52074 Aachen, Germany; Department of Neurology, Charité – Universitätsmedizin Berlin, Charité Campus Mitte, Charitéplatz 1, D-10117 Berlin, Germany; Department of Systems Neuroscience, University Medical Center Hamburg-Eppendorf, Martinistrasse 52, D-20246 Hamburg, Germany; Janssen Research & Development, L.L.C., 3210 Merryfield Row, San Diego, CA 92121 USA

**Keywords:** Transient receptor potential vanilloid 1, Capsaicin, Calcitonin gene-related peptide, Inflammatory soup, *c-fos*, Trigeminal activation

## Abstract

**Background:**

The capsaicin and heat responsive ion channel TRPV1 is expressed on trigeminal nociceptive neurons and has been implicated in the pathophysiology of migraine attacks. Here we investigate the efficacy of two TRPV1 channel antagonists in blocking trigeminal activation using two *in vivo* models of migraine.

**Methods:**

Male Sprague–Dawley rats were used to study the effects of the TRPV1 antagonists JNJ-38893777 and JNJ-17203212 on trigeminal activation. Expression of the immediate early gene *c-fos* was measured following intracisternal application of inflammatory soup. In a second model, CGRP release into the external jugular vein was determined following injection of capsaicin into the carotid artery.

**Results:**

Inflammatory up-regulation of *c-fos* in the trigeminal brain stem complex was dose-dependently and significantly reduced by both TRPV1 antagonists. Capsaicin-induced CGRP release was attenuated by JNJ-38893777 only in higher dosage. JNJ-17203212 was effective in all doses and fully abolished CGRP release in a time and dose-dependent manner.

**Conclusion:**

Our results describe two TRPV1 antagonists that are effective in two *in vivo* models of migraine. These results suggest that TRPV1 may play a role in the pathophysiological mechanisms, which are relevant to migraine.

## Background

Migraine is one of the most common debilitating disorders, affecting 324 million people worldwide [1]. Even though the acute treatment of migraine has greatly improved with the development of 5-HT_1B/1D_ receptor agonists (triptans), a substantial percentage of patients do not benefit from oral triptan formulations [2, 3]. Triptans are contraindicated in patients with cerebrovascular disease, cardiovascular disease, poorly controlled hypertension, severe hepatic or renal impairment and certain forms of migraine; triptans may also induce serotonin syndrome when taken in combination with selective serotonin-reuptake inhibitors [4]. In the majority of patients, the migraine attack is accompanied by cutaneous allodynia [5] and this phenomenon has been reported to be associated with a lack of efficacy of triptans [6]. Even though these findings were not supported by later studies [7–9], they emphasize the importance of developing alternative treatment options for patients that do not benefit from triptans.

The neurotransmitter calcitonin gene-related peptide (CGRP) plays a crucial role in the pathophysiology of migraine. Infusion of CGRP causes migraine-like disorders or even migraine without aura [10], and several CGRP receptor antagonists have been shown to be effective in the acute treatment of migraine [11–13]. It is well known that activation of the trigeminal nerve system induces a release of CGRP [14] and our group has recently demonstrated that this release could be almost completely abolished by destroying primary trigeminal afferents with neonatal capsaicin treatment [15].

Capsaicin activates the heat and pH-sensitive ion channel Transient Receptor Potential Vanilloid 1 (TRPV1), which seems to be involved in the pathophysiology of migraine [15–18]. TRPV1 is expressed on trigeminal nociceptors [19, 20], which innervate the dura mater and the meningeal vascular system [21]. Activation of TRPV1 causes release of CGRP from trigeminal nerve terminals [22–24] and neurogenic inflammation within the meninges [25], possibly initiating migraine attacks. Accordingly, the anti-migraine drug sumatriptan was recently shown to block trigeminal TRPV1 channels [26].

However, the effectiveness of specific antagonism of the TRPV1 channel in the treatment of migraine remains unresolved. Even though one antagonist, SB-705498, was shown to suppress and reverse sensitization upon dural inflammation [27], a clinical trial testing the compound in migraine patients has been terminated early due to lack of efficacy [28]. Another TRPV1 antagonist, A-993610, was recently shown to be ineffective in different animal models of migraine [29]. Here, we investigate the effectiveness of two TRPV1 receptor antagonists in blocking trigeminal activation, as measured by expression of the immediate early gene *c-fos* [30] and in preventing CGRP release, both common *in vivo* models of migraine.

## Methods

All experiments were approved by the local authorities, the Landesamt für Gesundheit und Soziales Berlin (Reg. 265/05). Animal procedures were conducted as previously described [15, 31, 32]. In short, male Sprague–Dawley rats (260–300 g, Charles-River, Sulzfeld, Germany) were anaesthetized with intraperitoneal (i.p.) thiopental-sodium (60 mg/kg body weight). Supplemental doses were administered during the experiments when necessary. Body temperature of the rats was maintained at 37 ± 0.5 °C using a heating blanket and a rectal probe. Rats were tracheotomized and mechanically ventilated with supplemental oxygen. Endexpiratory CO_2_ was continuously monitored (EGM 1, Heyer, Bad Ems, Germany). The femoral artery and vein were cannulated (Portex Polythene Tubing PE 50, neoLab GmbH, Heidelberg, Germany) for blood pressure monitoring and intravenous (i.v.) administration of compounds, respectively.

### Physiological variables

Mean arterial blood pressure, body temperature and arterial oxygen tension were measured continuously during the experiments. We analyzed data for a period of 2 min before the start of the infusion of the compounds and for 2 min at the end of the infusion. Sumatriptan was administered subcutaneously (s.c.) and in this group we measured physiological variables 20 min after the administration.

### c-fos study

A soft and flexible catheter (PE 0.28 mm i.d.) was introduced into the cisterna magna for administration of inflammatory soup (IS) as described elsewhere [15, 33]. Twenty minutes later, sumatriptan (s.c.; 300 μg/kg), JNJ TRPV1 antagonists or JNJ-vehicle (both i.v.) were administered over a period of 20 min. Immediately afterwards, 70 μl IS or IS-vehicle (0.9 % NaCl) was applied slowly over a period of 2 min using a 100 μl Hamilton syringe. Two hours after IS application, animals were given a sublethal dose of thiopental-sodium (100 mg/kg, i.p.) and then transcardially perfused with 50 ml saline and 330 ml of cold 4 % paraformaldehyde (PFA; in 0.1 M phosphate buffered saline (PBS); pH 7.4). The brain and cervical spinal cord were removed and the dura mater was assessed for damage or bleeding. If blood was found on the dura covering the hemispheres or the dura was destroyed, brains were not further processed. Brains were stored in fixative (4 % PFA in 0.1 M PBS; pH 7.4) overnight, followed by a cryoprotective solution (20 % sucrose + 0.5 % sodium azide) for another 24 h (both at 4 °C) and then further processed for *c-fos* staining as previously described [31]. Five to seven animals were used in each group with the exception of the groups that received the lowest doses of TRPV1 antagonist (0.03 mg/kg for JNJ-38893777 and 0.3 mg/kg for JNJ-17203212), in which only 3 animals were used.

*C-fos* like immunoreactive (*c-fos* LI) nuclei in the trigeminal brain stem complex were identified under bright field microscopy and counted by an observer naïve to the treatment as previously described [31, 34]. Cell were counted on both sides of the trigeminal brain stem complex. In control groups, we used animals that were instrumented in an identical way but received vehicle instead of inflammatory soup.

### CGRP study

The external jugular vein and carotid artery were cannulated (PE 0.86 mm i.d., PE 50, respectively). The catheter in the jugular vein was flushed with a mixture of heparin and NaCl to avoid clotting. After 30 min, a blood sample (500 μl) was taken from the jugular vein to determine the baseline CGRP concentration. Afterwards, sumatriptan (300 μg/kg body weight), JNJ TRPV1 antagonists or JNJ-vehicle were injected over a period of 20 min. At the end of the infusion, capsaicin (4 μmol/kg) or capsaicin-vehicle (saline : ethanol : Tween-80 in a ratio 8 : 1 : 1) was injected slowly over a period of 2 min into the carotid artery. Blood samples for CGRP level determination were taken after 5, 10, and 15 min as previously described [32]. Six to eight animals were used for each group.

### Drugs

Thiopental-sodium (Trapanal®) was purchased from Altana, Wesel, Germany. Capsaicin, EDTA disodium salt solution and Aprotinin (Aprotinin from bovine lung, 0.55 trypsin inhibitor units/ml blood) were purchased from Sigma, Steinheim, Germany. Sumatriptan (Imigran®) was purchased from GlaxoSmithKline, Uxbridge, UK. Ten-fold concentrated IS (histamine, serotonin, both 10 mM; bradykinin, prostaglandin E_2_, both 1 mM; pH 5.5; adapted from [35]) was formulated in PBS. JNJ-17203212 (4-(3-trifluoromethyl-pyridin-2-yl)-piperazine-1-carboxylic acid (5-trifluoromethyl-pyridin-2-yl)-amide) and JNJ-38893777 (2 (1 piperidinyl) N [4 (trifluoromethyl)phenyl] 7 [3 (trifluoromethyl) 2 pyridinyl] 6,7,8,9 tetrahydro 5H pyrimido[4,5 d]azepin 4 amine) were provided by Janssen Research & Development, L.L.C., and formulated in 5 % Pharmasolve, 20 % Cremophor, 75 % dextrose solution (5 % dextrose in water). The vehicle for the JNJ TRPV1 antagonists (“JNJ-vehicle”) consisted of the latter three substances in the same concentrations.

### Statistical analysis

Data were tested for normality using the Kolmogorov-Smirnov test, followed by one-way ANOVA and either Bonferroni correction (for CGRP study) or Dunnett’s test (for *c-fos* study) as *post-hoc* analysis for multiple comparisons. All values were normally distributed. Statistical significance was assumed when *p* < 0.05. All data are shown as mean ± SEM. Statistical analysis was performed using STATISTICA, version 8.0 (StatSoft, Inc. 2008).

## Results

### Physiological variables

Although there was a trend toward reduced mean arterial blood pressure in all but the vehicle groups, we did not see any significant changes in physiological variables. Temperature was slightly elevated during and shortly after JNJ TRPV1 antagonist infusion but changed back to baseline after a little more than 60 min. However, none of these effects were statistically significant.

### C-fos LI within TNC

#### IS induces an increase in c-fos LI within the trigeminal brain stem complex

**Fig. 1:**
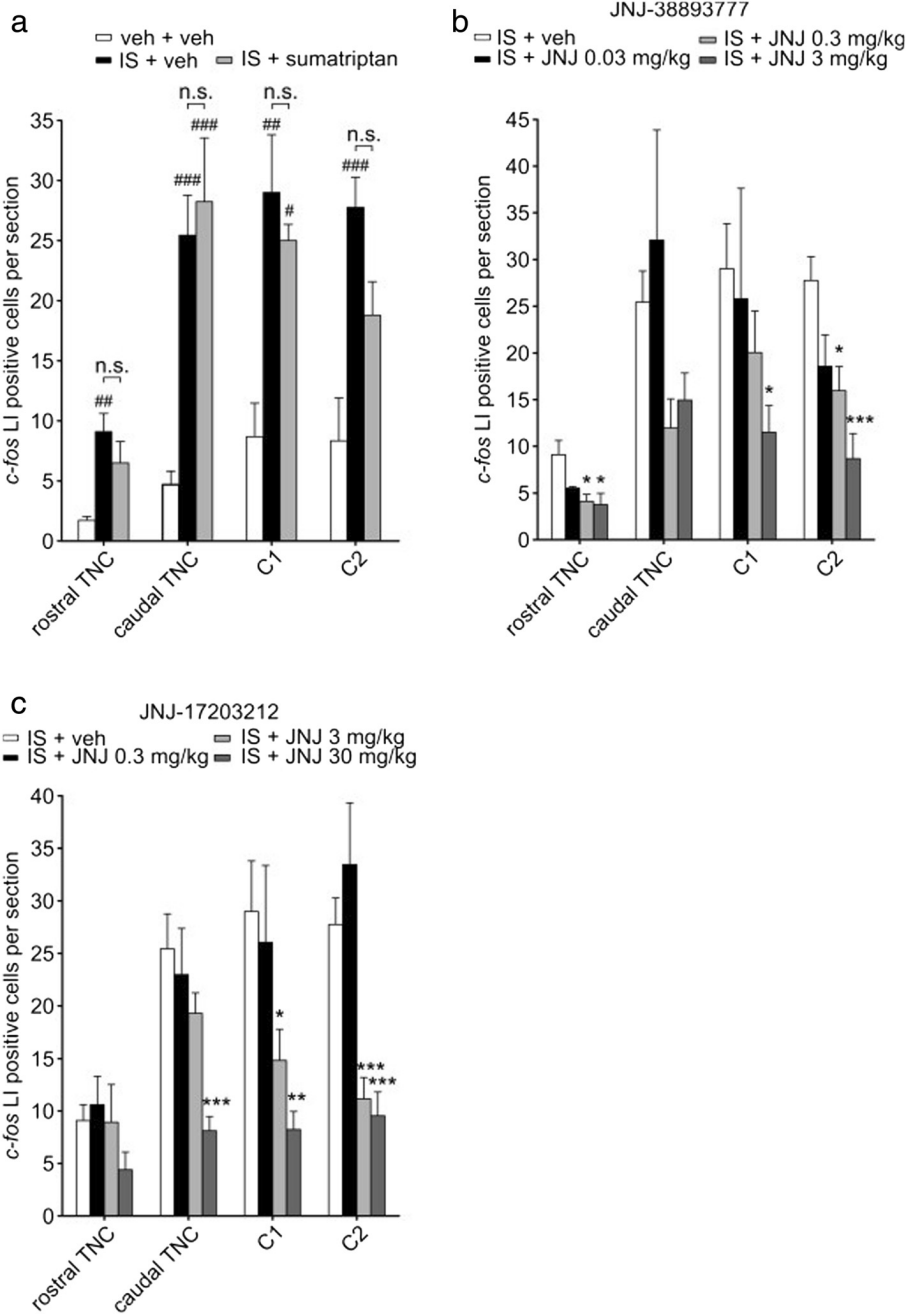
IS-induced up-regulation of *c-fos* can be abolished through block of TRPV1. **a** Intracisternal administration of inflammatory soup (IS; black bars) induces an increase in the amount of cells showing *c-fos* like immunoreactivity (*c-fos* LI) in the trigeminal brain stem complex. This increase was significant at all levels of the TNC when compared to control animals (white bars), injected with an identical volume of vehicle. These two groups also received an injection of sumatriptan vehicle. Administration of sumatriptan (300 μg/kg; grey bars) before injection of IS did not affect the IS-induced up-regulation of *c-fos*. **b** The TRPV1 antagonist JNJ-38893777 reduced the elevated *c-fos* expression, seen after injection of IS, in a dose-dependent fashion. The antagonist was injected intravenously in 0.03 mg/kg (black bars), 0.3 mg/kg (light grey bars) and 3 mg/kg (dark grey bars) over a period of 20 min before application of IS. **c** The TRPV1 antagonist JNJ-17203212 was injected in 0.3 mg/kg (black bars), 3 mg/kg (light grey bars) and 30 mg/kg (dark grey bars) previous to IS administration. A dose-dependent reduction in IS-induced *c-fos* expression could be observed. The highest dose completely abolished *c-fos* up-regulation in most parts of the TNC. # compares to vehicle + vehicle treated group (no IS administration) at the same level of TNC. * compares to IS + vehicle treated group at the same level of TNC. n.s. not significant (compared to IS + vehicle). *p < 0.05; **p < 0.01; ***p < 0.001

*C-fos* LI was mainly found in laminae 1 and 2 of the trigeminal nucleus caudalis (TNC) and was most pronounced in the lower levels, corresponding to C1. Only a few cells were seen in all other laminae, in accordance with previously published data [31, 33]. Intracisternal injection of 10-fold concentrated IS (10 mM histamine, serotonin; 1 mM bradykinin, prostaglandin E_2_; pH 5.5; adapted from [35]) resulted in an increased expression of *c-fos* within the TNC as compared to controls, which were exposed to an identical volume of vehicle for the same duration (Fig. 1a). After IS stimulation, we counted 9.1 ± 1.5 cells per section at the rostral level of the TNC (*p* = 0.002), 25.4 ± 3.3 cells in the caudal TNC (*p* < 0.001), 29 ± 4.8 cells at the C1 level (*p* = 0.002), and 27.8 ± 2.5 cells at the C2 level (*p* < 0.001, *n* = 7). For comparison, in animals treated with vehicle, we only counted 1.7 ± 0.3 in the rostral and 4.7 ± 1.1 in the caudal TNC, 8.7 ± 2.8 and 8.3 ± 3.6 at C1 and C2, respectively (*n* = 6).

Subcutaneous injection of sumatriptan had no significant effect on the IS-induced *c-fos* up-regulation (Fig. 1a; 6.5 ± 1.8 in the rostral TNC, 28.2 ± 5.3 in the caudal TNC, 25 ± 1.3 at C1, 18.8 ± 2.7 at C2; *p* ≥ 0.18 compared to IS + vehicle at all levels, ANOVA with Bonferroni correction; *n* = 5).

### JNJ-38893777 attenuates IS-induced c-fos LI

Treatment with the TRPV1 antagonist JNJ-38893777 led to a dose dependent reduction of IS-induced *c-fos* up-regulation in the rostral part as well as within levels C1 and C2 of the TNC (Fig. 1b). The lowest dose of 0.03 mg/kg did not show a significant effect at any level when compared to injection of IS plus vehicle (*p* ≥ 0.15). In contrast, at a 10-fold higher concentration, JNJ-38893777 significantly reduced the IS-induced up-regulation of *c-fos* LI in the rostral TNC (4.1 ± 0.8, *p =* 0.023) and at the level of the C2 (16 ± 2.6, *p =* 0.021). In the caudal TNC and the C1, a strong decrease in *c-fos* LI that was short of significance could be observed (11.9 ± 3.1, *p* = 0.072 and 20.05 ± 4.5, *p* = 0.433; *n* = 6). The highest dose of 3 mg/kg induced a significant reduction in *c-fos* LI in the rostral part of the TNC (3.8 ± 1.2, *p =* 0.011) and at the C1 level (11.5 ± 2.8, *p =* 0.036) and fully abolished the IS-induced increase of *c-fos* LI at the C2 level (8.7 ± 2.7, *p* < 0.001; *n* = 7). In the caudal part of the TNC we counted 14.9 ± 2.9 cells per section, which failed to reach significance when compared to IS plus vehicle (*p* = 0.17; *n* = 7).

### JNJ-17203212 dose-dependently reduces IS-induced c-fos expression

The TRPV1 antagonist JNJ-17203212 also had a dose-dependent effect on the elevated *c-fos* expression that occurred after intracisternal injection of IS (Fig. 1c). While the lowest dose failed to reduce the effect of IS (*p* ≥ 0.46), JNJ-17203212 at a concentration of 3 mg/kg significantly attenuated the IS-induced *c-fos* expression at the levels C1 and C2 of the TNC (14.8 ± 2.9, *p* = 0.038 at C1 and 11.2 ± 2, *p* < 0.001 at C2). A minimal reduction of *c-fos* LI in comparison to the control group could be observed in the caudal TNC for this dose (19.3 ± 2, *p* = 0.24), whereas no effect could be observed in more rostral parts of the TNC (8.9 ± 3.6, *p* = 0.99; *n* = 7). JNJ-17203212 at the highest concentration of 30 mg/kg completely abolished *c-fos* up-regulation in the caudal TNC (8.1 ± 1.4, *p* < 0.001) as well as at levels C1 (8.3 ± 1.7, *p =* 0.005) and C2 of the TNC (9.6 ± 2.3, *p* < 0.001; *n* = 5).

### CGRP study

Next, we measured the effect of both TRPV1 antagonists on the release of CGRP into jugular vein blood induced by the common TRPV1 agonist capsaicin. While this is itself a common *in vivo* model of migraine, it would also provide proof for target engagement by the two antagonists and could thus confirm that the above results in IS-induced *c-fos* expression are most likely due to block of TRPV1.

### Capsaicin leads to elevated CGRP levels in jugular vein blood

**Fig. 2:**
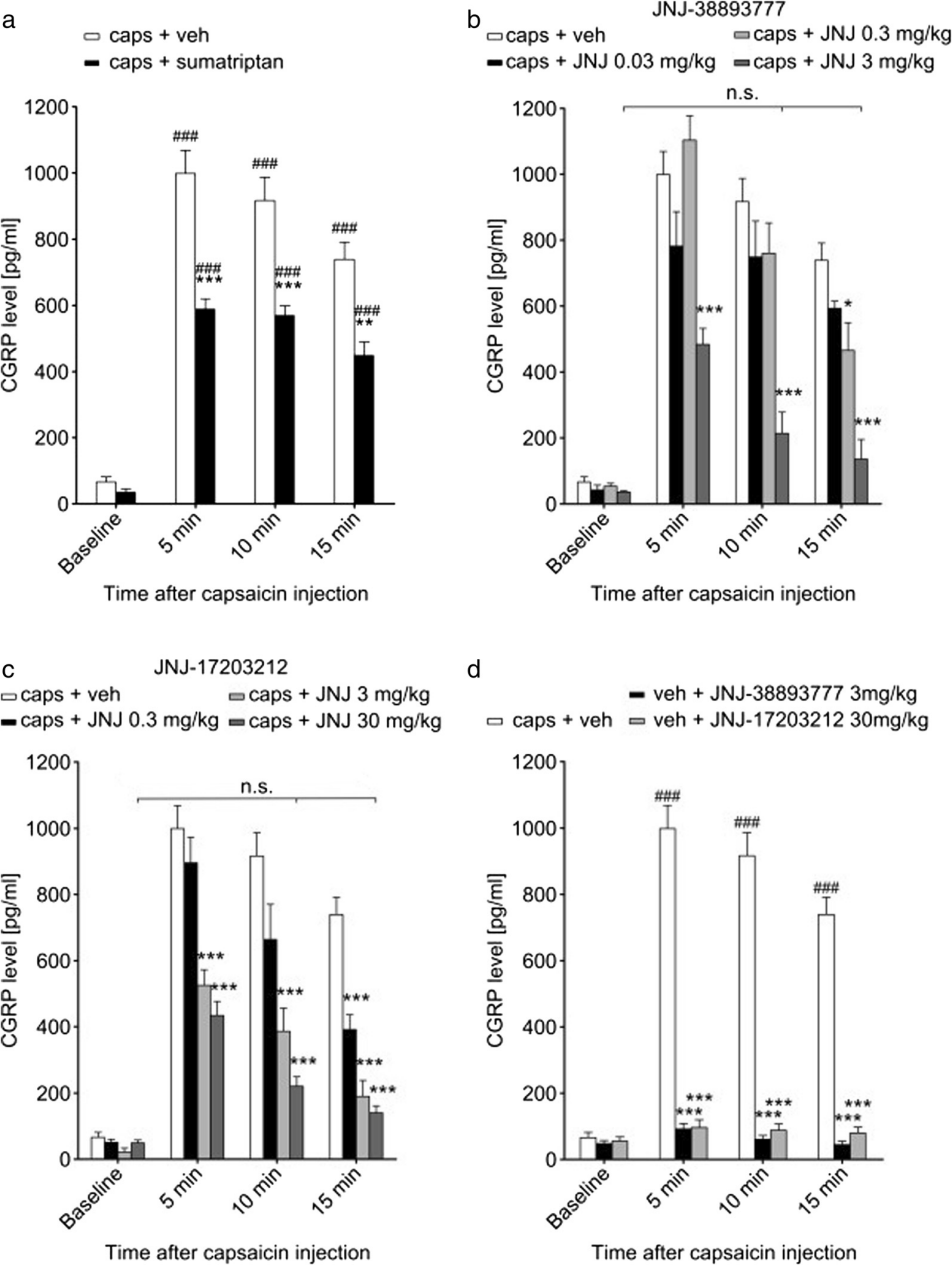
TRPV1 antagonists are effective in blocking capsaicin-induced CGRP release. **a** Injection of capsaicin (4 μmol/kg) into the carotid artery caused an increase in CGRP concentration in jugular vein blood. The increase was substantial within 5 min and still significant albeit slightly decreasing after 15 min. Administration of sumatriptan (300 μg/kg; black bars) prior to capsaicin treatment reduced the elevated CGRP levels significantly, although they were still considerably higher than baseline values. **b** The TRPV1 antagonist JNJ-38893777 in the highest dose of 3 mg/kg (dark grey bars) reduced jugular CGRP levels after capsaicin significantly, returning them to near baseline levels within 10 min. Lower doses of 0.3 mg/kg (light grey bars) and 0.03 mg/kg (black bars) were less effective. **c** JNJ-17203212 had a dose-dependent effect on the capsaicin-induced increase in jugular CGRP concentration. CGRP release was completely abolished within 10 min when the antagonist was administered at 30 mg/kg (dark grey bars) and within 15 min when administered at 3 mg/kg (light grey bars). The lowest dose of 0.3 mg/kg (black bars) reduced the CGRP levels significantly but not to the level of the pre-capsaicin baseline after 15 min. **d** Neither JNJ-38893777 (black bars) nor JNJ-17203212 (grey bars) when used in their highest doses had any effect on jugular CGRP levels in animals that did not receive an injection of capsaicin. Values were not significantly different compared to the respective baseline. # compares to baseline values of the same group. * compares to capsaicin + vehicle at the same time point. n.s. not significant (compared to baseline values of the same group). *p < 0.05; **p < 0.01; ***p < 0.001

Baseline plasma CGRP levels in jugular vein blood were not different between any of the groups. In the control group treated intravenously with vehicle, we measured a baseline CGRP concentration of 65.7 ± 15.7 pg/ml. Injection of capsaicin (4 μmol/kg) into the carotid artery caused a significant increase in CGRP blood levels that was sustained for 15 min (Fig. 2a). We measured a CGRP concentration of 998.9 ± 69.9 pg/ml 5 min after capsaicin administration, 916.5 ± 70.5 pg/ml after 10 min and 738.8 ± 52.4 pg/ml after 15 min (*n* = 9, *p* < 0.001 at all time points). To demonstrate that the capsaicin-induced increase in CGRP blood levels was mediated via the trigeminal nerve system, we administered the common migraine drug sumatriptan (300 μg/kg) subcutaneously, prior to capsaicin treatment. Sumatriptan significantly reduced the increase in jugular CGRP levels (Fig. 2a). In this group, we measured only 589.5 ± 30.1 pg/ml 5 min after capsaicin administration, 570.2 ± 28 pg/ml after 10 min (both *p* < 0.001) and 450.1 ± 39 pg/ml (*p* = 0.002; *n* = 8) after 15 min.

### JNJ-38893777 reduces capsaicin-induced CGRP release

The TRPV1 antagonist JNJ-38893777 significantly reduced the increase in jugular CGRP levels that could be measured 5 min after capsaicin injection and completely abolished the increase measured after 10 and 15 min when administered at 3 mg/kg (Fig. 2b; 483.4 ± 48.5 pg/ml, 213.7 ± 64.7 pg/ml, and 135.7 ± 59.3 pg/ml after 5, 10, and 15 min, respectively, *p* < 0.001 at all time points). At a 10-fold lower dose, the antagonist was only effective at the 15 min time point (465.1 ± 84.3 pg/ml, *p* = 0.013) but showed no effect earlier than that. At the lowest dose of 0.03 mg/kg, JNJ-38893777 had no effect (*n* = 7 for all groups).

### JNJ-17203212 completely blocks capsaicin-induced CGRP release in a dose-dependent manner

Treatment with the TRPV1 antagonist JNJ-17203212 had a dose dependent effect on capsaicin-induced CGRP release (Fig. 2c). Five minutes after capsaicin application, the groups treated with the two higher doses of 3 mg/kg (525 pg/ml ± 47.4 pg/ml, *p* < 0.001, *n* = 7) and 30 mg/kg (433.9 pg/ml ± 42.9 pg/ml, *p* < 0.001, *n* = 7) showed significantly different CGRP levels compared to the capsaicin plus vehicle treated group. At the 10 min time point, the lowest dose of 0.3 mg/kg (664.4 ± 105.8 pg/ml, *p* = 0.13, *n* = 8) showed a strong tendency towards decreased jugular CGRP levels but this effect did not reach significance. At 3 mg/kg, CGRP release was significantly reduced (386.2 ± 70.3 pg/ml, *p* < 0.001) and at 30 mg/kg, it was completely abolished (220.7 ± 28.7 pg/ml; *p* < 0.001, but *p* = 0.72 compared to baseline from the same group). After 15 min, all three doses of JNJ-17203212 induced significant effects when compared to the capsaicin plus vehicle treated group (391.4 ± 45.4 pg/ml, 190 ± 48.3 pg/ml, 140.4 ± 21 pg/ml for 5, 10, and 15 min, resp., *p* < 0.001 for all doses). At this time point, the two higher doses fully abolished the capsaicin-induced CGRP release.

None of the antagonists when used in their highest dose had any effect on jugular CGRP levels in animals that were treated with capsaicin-vehicle (Fig. 2d; *n* = 7).

## Discussion

In this study we applied two *in vivo* models of migraine to test the efficacy of two TRPV1 antagonists. It is commonly believed that activation of the trigeminal nerve system and subsequent release of CGRP from trigeminal fibres, which leads to vasodilation within the meninges, plays a crucial role in the development of migraine attacks. This is supported by the fact that several CGRP receptor antagonists are effective in the acute treatment of migraine [11–13]. We have shown here that antagonists of the TRPV1 ion channel could be effective in blocking this trigeminal activation that leads to the release of CGRP.

First, we tested the effect of two TRPV1 antagonists on the stimulus-induced up-regulation of the immediate early gene *c-fos* in the trigeminal brain stem complex. We selected the so-called inflammatory soup (IS) as activating stimulus, which is a well-established model to study the mechanisms underlying the sensitization of trigeminal primary afferent neurons. This central sensitization has been considered to be the cause of certain headaches and is almost certainly the underlying mechanism for allodynia, which often accompanies migraine attacks [36, 37]. Our group has shown that IS activates the trigeminal nerve system when administered intracisternally as demonstrated by CGRP release [32]. Here, we confirm these findings by showing that intracisternal administration of IS leads to a pronounced up-regulation of *c-fos* LI within the TNC. The amount of *c-fos* expression in response to IS was found to be considerably less than what has previously been reported as the response to capsaicin [31]. As IS predominantly acts through the sensitization rather than direct activation of nociceptive ion channels, this result was not unexpected. Elevated *c-fos* in the TNC after stimulation of the dura with IS has been reported previously and the results correspond well with the findings of this study [38, 39]. However, Edelmayer et al. [39] have shown sumatriptan to be effective in blocking *c-fos* up-regulation, which does not correspond to our results. This discrepancy is likely due to lower concentration of inflammatory mediators and higher concentration of sumatriptan used by Edelmayer et al. We have shown in this and earlier reports that the lower sumatriptan dose administered here is already effective as it blocks both capsaicin and IS-induced CGRP release [32]. We do not propose a direct effect of sumatriptan on TRPV1 channels but rather an inhibition of CGRP release by the drug. However, sumatriptan does not reverse sensitization of already sensitized central trigeminal neurons [40], thus explaining the absence of an effect on the IS-induced *c-fos* up-regulation in this study. The TRPV1 antagonist JNJ-38893777 in its highest dose (3 mg/kg) decreased the IS-induced *c-fos* LI significantly, in some areas even to the level of control animals not treated with IS. The second antagonist JNJ-17203212 showed remarkable efficacy in all levels of the TNC at 30 mg/kg, completely abolishing *c-fos* up-regulation. To corroborate the above results, we tested the two TRPV1 antagonists for their ability to block stimulus-induced CGRP release into jugular vein blood. Capsaicin was used as the stimulus as this substance has been used in many preclinical migraine drug studies previously [41, 42]. In addition, the use of capsaicin allowed us to demonstrate the efficacy of the two antagonists in blocking the TRPV1 channel. Injection of capsaicin into the carotid artery caused a significant increase in jugular CGRP levels that was sustained for 15 min. The TRPV1 antagonist JNJ-38893777 at 3 mg/kg was more effective than sumatriptan and returned CGRP levels close to baseline. The TRPV1 antagonist JNJ-17203212 was effective in reducing capsaicin-induced CGRP release at all three concentrations. These results demonstrate the specificity of the two compounds against the TRPV1 channel and suggest that the IS-induced up-regulation of *c-fos* was also due to a block of TRPV1.

The specificity of the two antagonists used in this study has been tested extensively *in vitro* and the results are summarized in Table 1. Both compounds were tested against a number of TRP channels as well as against a broad panel of other non-related receptors and channels. JNJ-17203212 displayed some weak inhibition of TRPM8 that remained far below the activity at the TRPV1 channel, but was not active at any of the other receptors and channels [43]. JNJ-38893777 was not active at any of the tested TRP channels but displayed some weak activity at the human Cholecystokinin 1 receptor (p*K*_i_ 6.1), the human Adenosine 3 receptor (p*K*_i_ 6.2) and a rat cerebral cortex sodium channel (p*K*_i_ 6.1). However, these activities were far lower compared to the inhibition of hTRPV1 (p*K*_i_ 8.0). In conclusion, while we cannot exclude the possibility that the effect of the two antagonists on *c-fos* expression could also be mediated by additional targets, a significant effect seems unlikely given the higher specificity of the compounds against the TRPV1 channel and the TRPV1-specific block observed in the CGRP experiments.

**Table 1:** Pharmacological selectivity of JNJ compounds

	JNJ-38893777	JNJ-17203212
pIC_50_ (capsaicin)	8.08	7.19
pIC_50_ (low pH)	8.13	7.8
p*K*_i_ (hTRPV1)	8.0	7.3
Activity against related TRP channels^a^	no activity (pIC_50_ < 5)	weak activity against cTRPM8 (pIC_50_ < 6)
Activity against non-related channels or receptors	weak activity against:	no activity
	human Cholecystokinin 1 receptor (p*K*_i_ 6.1)	
	human Adenosine 3 receptor (p*K*_i_ 6.2)	
	rat cerebral cortex sodium channel (p*K*_i_ 6.1)	

pIC_50_ values were determined using the recombinant hTRPV1 in a Ca^2+^ influx *in vitro* assay (FLIPR) [58]. p*K*_i_ values were determined by radioligand binding to a broad panel of receptors, channels and transporters at CEREP (Paris, France) [43]

^a^Activity was tested against the following related TRP channels: rTRPV2, hTRPV4, hTRPA1, cTRPM8

TRPV1 is well known for its role in nociception and sensitization and has been widely studied in the peripheral nociceptive system [44]. In the central nervous system, TRPV1 expression has been shown in the TNC and in numerous other areas [26, 45–47], many of which are involved in the processing during headaches [48]. TRPV1 is expressed by a majority of CGRP-releasing trigeminal fibres that innervate the meningeal vascular system [15, 49]. Stimulation of these trigeminal afferents through activation of TRPV1 causes a CGRP-mediated increase in dural blood flow [22, 24, 49] and activation of second order neurons in the TNC [33], and these mechanisms could be involved in the initiation of migraine attacks. Because activation of the trigeminal nucleus is essential during the pain phase of a migraine attack, we decided to study the effect of TRPV1 antagonism in this anatomical region. While we cannot exclude the possibility that the TRPV1 antagonists used in this study also acted on sites other than the TNC, our *c-fos* data point to an efficacy of the two compounds in that particular area. This is in good agreement with previous data, showing that functional antagonism at the TRPV1 receptor may modulate neurotransmission in the TNC [50].

Research on the importance of the TRPV1 channel in the pathophysiology of migraine is, however, inconclusive. A growing body of evidence seems to support a role of TRP channels in general and TRPV1 in particular in the pathomechanisms of headaches [16, 51]. A recent study investigating single nucleotide polymorphisms among the Spanish population identified the TRPV1 and TRPV3 genes as likely candidates for contributing to an increased genetic susceptibility to migraine [17]. The well-known migraine trigger ethanol has been shown to induce neurogenic vasodilation via a TRPV1-mediated release of CGRP [24]. Furthermore, TRPV1 channels in the TNC or in dissociated trigeminal neurons were shown to be inhibited by the common migraine drug sumatriptan [26]. This is in line with the findings in this study that show reduced trigeminal activation upon block of TRPV1 channels. Confirming these observations, electrophysiological *in vivo* studies have shown that a direct inhibition of the TRPV1 channel using the TRPV1 antagonist SB-705498, as well as the functional antagonism using the TRPV1 agonist olvanil, which induces a long-lasting neuronal desensitization, suppress neuronal activity in the trigeminocervical complex following nociceptive dural stimulation [27, 50]. A recent study showed that CGRP increases TRPV1 expression in the trigeminal nociceptive system [18]. This is strengthened by the observation that CGRP levels in jugular vein blood of human patients are elevated during migraine attacks [52]. Taken together, these findings emphasize the importance of the interplay of TRPV1 and CGRP in migraine-related processes. However, the interpretation of these findings is still controversial as conflicting data exists. In this context, electrophysiological studies using another TRPV1 antagonist, A-993610, failed to show alleviating effects in different animal models of migraine [29]. The authors measured trigeminal firing, induced by electrical stimulation of the middle meningeal artery, neurogenic dural vasodilation and mechanically induced cortical spreading depression. The failure of A-993610 to be effective is in contrast to the results of this study. We used two different models of trigeminal activation and two completely different TRPV1 antagonists, both of which showed significant effects. The published IC_50_ value for A-993610 [53] seems to lie in a similar range compared to the two compounds used here. However, no further information on pharmacokinetics or bioavailability for this particular compound could be found. In light of the promising data reported here, it seems that it might be warranted to test the two compounds described here in an experimental paradigm similar to that described by Summ et al. [29] as well as in further clinical studies.

A major problem with the clinical use of early developed TRPV1 antagonists was a significant hyperthermia, which in several human patients lasted for several days and could exceed 40 °C [54, 55]. However, more recently developed antagonists have been shown to avoid this severe side effect [56]. Nevertheless, the use of TRPV1 antagonists for the treatment of acute headache or migraine is controversial. The promising TRPV1 antagonist SB-705498 was shown to be effective in preventing and reversing sensitization of responses to electrical stimulation, induced by topical application of IS onto the dura mater of cats [27]. Conflicting results were, however, obtained in human patients. A clinical trial using this compound to treat acute migraine has been terminated early due to a lack of efficacy [28]. SB-705498 had previously failed in reducing capsaicin-evoked hyperalgesia and had only minimal effects on capsaicin-induced flare [57], and it is not clear if the properties of this compound are representative of all TRPV1 antagonists. Direct comparison revealed an overall higher TRPV1 affinity of the compounds used in this study compared to SB-705498. Both JNJ compounds have higher p*K*_i_ and in some paradigms higher pIC_50_ values [43]. JNJ-17203212 also displays a longer half-life of 7.4 h [58] compared to 3.1 h for SB-705498 [59]. Furthermore, oral bioavailability exceeds that of SB-705498 by 14 %. JNJ-38893777 was recently shown in a human study to be well tolerated without causing serious adverse events and to be suitable for further clinical development [60]. Taken together, these findings might suggest that the compound SB-705498 and the dosage used in the previous clinical trial were not optimal. Additional clinical studies with other TRPV1 antagonists should therefore be conducted before definitive conclusions on the role of TRPV1 in migraine can be drawn.

## Conclusion

Our results describe two TRPV1 antagonists that are effective in two *in vivo* models of migraine. We have shown that block of the TRPV1 receptor can be effective in inhibiting trigeminal activation, as seen by the reduced expression of *c-fos* in the TNC. While for JNJ-38893777 the effects were not as clear as for JNJ-17203212, both antagonists still seem to be promising candidates for additional studies to further characterize their efficacy using different animal models of migraine pain. Based on our findings, we conclude that TRPV1 may play a role in the pathophysiological mechanisms, which are relevant to migraine.

## References

[1] World Health Organization (2008). The global burden of disease: 2004 update.

[2] Ferrari MD, Roon KI, Lipton RB, Goadsby PJ (2001). Oral triptans (serotonin 5-HT(1B/1D) agonists) in acute migraine treatment: a meta-analysis of 53 trials. Lancet.

[3] Lipton R, Buse D, Serrano D, Ng-Mak D, Pearlman S, Reed M (2011). Examination of unmet treatment needs among persons with episodic migraine (EM): Results of the American Migraine Prevalence and Prevention Study (AMPP) (P1342). Eur J Neurol.

[4] Loder E (2010). Triptan therapy in migraine. N Engl J Med.

[5] Lipton RB, Bigal ME, Ashina S, Burstein R, Silberstein S, Reed ML, Serrano D, Stewart WF (2008). Cutaneous allodynia in the migraine population. Ann Neurol.

[6] Burstein R, Collins B, Jakubowski M (2004). Defeating migraine pain with triptans: A race against the development of cutaneous allodynia. Ann Neurol.

[7] Cady R, Martin V, Mauskop A, Rodgers A, Hustad CM, Ramsey KE, Skobieranda F (2007). Symptoms of cutaneous sensitivity pre-treatment and post-treatment: results from the rizatriptan TAME studies. Cephalalgia.

[8] Goadsby PJ, Zanchin G, Geraud G, de Klippel N, Diaz-Insa S, Gobel H, Cunha L, Ivanoff N, Falques M, Fortea J (2008). Early vs. non-early intervention in acute migraine — “Act When Mild (AwM)”. A double-blind, placebo-controlled trial of almotriptan. Cephalalgia.

[9] Schoenen J, Klippel ND, Giurgea S, Herroelen L, Jacquy J, Louis P, Monseu G, Vandenheede M (2008). Almotriptan and its combination with aceclofenac for migraine attacks: a study of efficacy and the influence of auto-evaluated brush allodynia. Cephalalgia.

[10] Lassen LH, Haderslev PA, Jacobsen VB, Iversen HK, Sperling B, Olesen J (2002). CGRP may play a causative role in migraine. Cephalalgia.

[11] Olesen J, Diener HC, Husstedt IW, Goadsby PJ, Hall D, Meier U, Pollentier S, Lesko LM (2004). Calcitonin gene-related peptide receptor antagonist BIBN 4096 BS for the acute treatment of migraine. N Engl J Med.

[12] Ho TW, Ferrari MD, Dodick DW, Galet V, Kost J, Fan X, Leibensperger H, Froman S, Assaid C, Lines C, Koppen H, Winner PK (2008). Efficacy and tolerability of MK-0974 (telcagepant), a new oral antagonist of calcitonin gene-related peptide receptor, compared with zolmitriptan for acute migraine: a randomised, placebo-controlled, parallel-treatment trial. Lancet.

[13] Diener H-C, Barbanti P, Dahlöf C, Reuter U, Habeck J, Podhorna J (2011). BI 44370 TA, an oral CGRP antagonist for the treatment of acute migraine attacks: results from a phase II study. Cephalalgia.

[14] Zagami AS, Goadsby PJ, Edvinsson L (1990). Stimulation of the superior sagittal sinus in the cat causes release of vasoactive peptides. Neuropeptides.

[15] Hoffmann J, Wecker S, Neeb L, Dirnagl U, Reuter U (2012). Primary trigeminal afferents are the main source for stimulus-induced CGRP release into jugular vein blood and CSF. Cephalalgia.

[16] Meents JE, Neeb L, Reuter U (2010). TRPV1 in migraine pathophysiology. Trends Mol Med.

[17] Carreño O, Corominas R, Fernández‐Morales J, Camiña M, Sobrido M, Fernández‐Fernández JM, Pozo‐Rosich P, Cormand B, Macaya A (2011). SNP variants within the vanilloid TRPV1 and TRPV3 receptor genes are associated with migraine in the Spanish population. Am J Med Genet B Neuropsychiatr Genet.

[18] Chatchaisak D, Srikiatkhachorn A, Grand SM, Govitrapong P, Chetsawang B (2013). The role of calcitonin gene-related peptide on the increase in transient receptor potential vanilloid-1 levels in trigeminal ganglion and trigeminal nucleus caudalis activation of rat. J Chem Neuroanat.

[19] Guo A, Vulchanova L, Wang J, Li X, Elde R (1999). Immunocytochemical localization of the vanilloid receptor 1 (VR1): relationship to neuropeptides, the P2X3 purinoceptor and IB4 binding sites. Eur J Neurosci.

[20] Bae YC, Oh JM, Hwang SJ, Shigenaga Y, Valtschanoff JG (2004). Expression of vanilloid receptor TRPV1 in the rat trigeminal sensory nuclei. J Comp Neurol.

[21] Edvinsson L (2001). Sensory nerves in man and their role in primary headaches. Cephalalgia.

[22] Akerman S, Kaube H, Goadsby PJ (2003). Vanilloid type 1 receptors (VR1) on trigeminal sensory nerve fibres play a minor role in neurogenic dural vasodilatation, and are involved in capsaicin-induced dural dilation. Br J Pharmacol.

[23] Akerman S, Kaube H, Goadsby PJ (2004). Anandamide acts as a vasodilator of dural blood vessels in vivo by activating TRPV1 receptors. Br J Pharmacol.

[24] Nicoletti P, Trevisani M, Manconi M, Gatti R, De SG, Zagli G, Benemei S, Capone JA, Geppetti P, Pini LA (2008). Ethanol causes neurogenic vasodilation by TRPV1 activation and CGRP release in the trigeminovascular system of the guinea pig. Cephalalgia.

[25] Williamson DJ, Hargreaves RJ (2001). Neurogenic inflammation in the context of migraine. Microsc Res Tech.

[26] Evans MS, Cheng X, Jeffry JA, Disney KE, Premkumar LS (2012). Sumatriptan inhibits TRPV1 channels in trigeminal neurons. Headache J Head Face Pain.

[27] Lambert GA, Davis JB, Appleby JM, Chizh BA, Hoskin KL, Zagami AS (2009). The effects of the TRPV1 receptor antagonist SB-705498 on trigeminovascular sensitisation and neurotransmission. Naunyn Schmiedebergs Arch Pharmacol.

[28] Chizh B, Palmer J, Lai R, Guillard F, Bullman J, Baines A, Napolitano A, Appleby J (2009). 702 A randomised, two-period cross-over study to investigate the efficacy of the Trpv1 antagonist SB-705498 in acute migraine. Eur J Pain.

[29] Summ O, Holland PR, Akerman S, Goadsby PJ (2011). TRPV1 receptor blockade is ineffective in different in vivo models of migraine. Cephalalgia.

[30] Mitsikostas DD, Sanchez del Rio M (2001). Receptor systems mediating c-fos expression within trigeminal nucleus caudalis in animal models of migraine. Brain Res Rev.

[31] Offenhauser N, Zinck T, Hoffmann J, Schiemann K, Schuh-Hofer S, Rohde W, Arnold G, Dirnagl U, Jansen-Olesen I, Reuter U (2005). CGRP release and C-Fos expression within trigeminal nucleus caudalis of the rat following glyceryltrinitrate infusion. Cephalalgia.

[32] Hoffmann J, Neeb L, Israel H, Dannenberg F, Triebe F, Dirnagl U, Reuter U (2009). Intracisternal injection of inflammatory soup activates the trigeminal nerve system. Cephalalgia.

[33] Mitsikostas DD, Sanchez del Rio M, Waeber C, Moskowitz MA, Cutrer FM (1998). The NMDA receptor antagonist MK-801 reduces capsaicin-induced c-fos expression within rat trigeminal nucleus caudalis. Pain.

[34] Strassman AM, Mineta Y, Vos BP (1994). Distribution of fos-like immunoreactivity in the medullary and upper cervical dorsal horn produced by stimulation of dural blood vessels in the rat. J Neurosci.

[35] Steen KH, Steen AE, Reeh PW (1995). A dominant role of acid pH in inflammatory excitation and sensitization of nociceptors in rat skin, in vitro. J Neurosci.

[36] Burstein R, Yamamura H, Malick A, Strassman AM (1998). Chemical stimulation of the intracranial dura induces enhanced responses to facial stimulation in brain stem trigeminal neurons. J Neurophysiol.

[37] Jakubowski M, Levy D, Kainz V, Zhang X-C, Kosaras B, Burstein R (2007). Sensitization of central trigeminovascular neurons: blockade by intravenous naproxen infusion. Neuroscience.

[38] Maneepak M, Le Grand S, Srikiatkhachorn A (2009). Serotonin depletion increases nociception-evoked trigeminal NMDA receptor phosphorylation. Headache J Head Face Pain.

[39] Edelmayer RM, Vanderah TW, Majuta L, Zhang E-T, Fioravanti B, De Felice M, Chichorro JG, Ossipov MH, King T, Lai J, Kori SH, Nelsen AC, Cannon KE, Heinricher MM, Porreca F (2009). Medullary pain facilitating neurons mediate allodynia in headache-related pain. Ann Neurol.

[40] Levy D, Jakubowski M, Burstein R (2004). Disruption of communication between peripheral and central trigeminovascular neurons mediates the antimigraine action of 5HT 1B/1D receptor agonists. Proc Natl Acad Sci USA.

[41] Kaube H, Limmroth V (1996). Tiermodelle und ihre Konsequenzen für die therapie der Migräne. Schmerz.

[42] Eltorp C, Jansen-Olesen I, Hansen A (2000). Release of Calcitonin Gene-Related Peptide (CGRP) from guinea pig dura mater in vitro is inhibited by sumatriptan but unaffected by nitric oxide. Cephalalgia.

[43] Bhattacharya A, Scott BP, Nasser N, Ao H, Maher MP, Dubin AE, Swanson DM, Shankley NP, Wickenden AD, Chaplan SR (2007). Pharmacology and antitussive efficacy of 4-(3-trifluoromethyl-pyridin-2-yl)-piperazine-1-carboxylic acid (5-Trifluoromethyl-pyridin-2-yl)-amide (JNJ17203212), a transient receptor potential vanilloid 1 antagonist in guinea pigs. J Pharmacol Exp Ther.

[44] Szallasi A, Cortright DN, Blum CA, Eid SR (2007). The vanilloid receptor TRPV1: 10 years from channel cloning to antagonist proof-of-concept. Nat Rev Drug Discov.

[45] Davies AJ, North RA (2009). Electrophysiological and morphological properties of neurons in the substantia gelatinosa of the mouse trigeminal subnucleus caudalis. Pain.

[46] Roberts JC, Davis JB, Benham CD (2004). [3H]Resiniferatoxin autoradiography in the CNS of wild-type and TRPV1 null mice defines TRPV1 (VR-1) protein distribution. Brain Res.

[47] Mezey E, Toth ZE, Cortright DN, Arzubi MK, Krause JE, Elde R, Guo A, Blumberg PM, Szallasi A (2000). Distribution of mRNA for vanilloid receptor subtype 1 (VR1), and VR1-like immunoreactivity, in the central nervous system of the rat and human. Proc Natl Acad Sci U S A.

[48] Goadsby PJ, Charbit AR, Andreou AP, Akerman S, Holland PR (2009). Neurobiology of migraine. Neuroscience.

[49] Dux M, Sántha P, Jancsó G (2003). Capsaicin-sensitive neurogenic sensory vasodilatation in the dura mater of the rat. J Physiol.

[50] Hoffmann J, Supronsinchai W, Andreou AP, Summ O, Akerman S, Goadsby PJ (2012). Olvanil acts on transient receptor potential vanilloid channel 1 and cannabinoid receptors to modulate neuronal transmission in the trigeminovascular system. Pain.

[51] Dux M, Sántha P, Jancsó G (2012). The role of chemosensitive afferent nerves and TRP ion channels in the pathomechanism of headaches. Pflüg Arch Eur J Physiol.

[52] Goadsby PJ, Edvinsson L, Ekman R (1990). Vasoactive peptide release in the extracerebral circulation of humans during migraine headache. Ann Neurol.

[53] Gavva NR, Treanor JJS, Garami A, Fang L, Surapaneni S, Akrami A, Alvarez F, Bak A, Darling M, Gore A, Jang GR, Kesslak JP, Ni L, Norman MH, Palluconi G, Rose MJ, Salfi M, Tan E, Romanovsky AA, Banfield C, Davar G (2008). Pharmacological blockade of the vanilloid receptor TRPV1 elicits marked hyperthermia in humans. Pain.

[54] Gavva NR, Bannon AW, Surapaneni S, Hovland DN, Lehto SG, Gore A, Juan T, Deng H, Han B, Klionsky L, Kuang R, Le A, Tamir R, Wang J, Youngblood B, Zhu D, Norman MH, Magal E, Treanor JJS, Louis J-C (2007). The vanilloid receptor TRPV1 is tonically activated in vivo and involved in body temperature regulation. J Neurosci.

[55] Lehto SG, Tamir R, Deng H, Klionsky L, Kuang R, Le A, Lee D, Louis J-C, Magal E, Manning BH, Rubino J, Surapaneni S, Tamayo N, Wang T, Wang J, Wang J, Wang W, Youngblood B, Zhang M, Zhu D, Norman MH, Gavva NR (2008). Antihyperalgesic effects of (R, E)-N-(2-Hydroxy-2,3-dihydro-1H-inden-4-yl)-3-(2-(piperidin-1-yl)-4-(trifluoromethyl)phenyl)-acrylamide (AMG8562), a novel transient receptor potential vanilloid type 1 modulator that does Not cause hyperthermia in rats. J Pharmacol Exp Ther.

[56] Chizh BA, O’Donnell MB, Napolitano A, Wang J, Brooke AC, Aylott MC, Bullman JN, Gray EJ, Lai RY, Williams PM, Appleby JM (2007). The effects of the TRPV1 antagonist SB-705498 on TRPV1 receptor-mediated activity and inflammatory hyperalgesia in humans. Pain.

[57] Swanson DM, Dubin AE, Shah C, Nasser N, Chang L, Dax SL, Jetter M, Breitenbucher JG, Liu C, Mazur C, Lord B, Gonzales L, Hoey K, Rizzolio M, Bogenstaetter M, Codd EE, Lee DH, Zhang S-P, Chaplan SR, Carruthers NI (2005). Identification and biological evaluation of 4-(3-Trifluoromethylpyridin-2-yl)piperazine-1-carboxylic acid (5-Trifluoromethylpyridin-2-yl)amide, a high affinity TRPV1 (VR1) Vanilloid Receptor Antagonist. J Med Chem.

[58] Gunthorpe MJ, Hannan SL, Smart D, Jerman JC, Arpino S, Smith GD, Brough S, Wright J, Egerton J, Lappin SC, Holland VA, Winborn K, Thompson M, Rami HK, Randall A, Davis JB (2007). Characterization of SB-705498, a potent and selective vanilloid receptor-1 (VR1/TRPV1) antagonist that inhibits the capsaicin-, acid-, and heat-mediated activation of the receptor. J Pharmacol Exp Ther.

[59] Manitpisitkul P, Mayorga A, Shalayda K, Meulder MD, Romano G, Jun C, Moyer JA (2015). Safety, tolerability and pharmacokinetic and pharmacodynamic learnings from a double-blind, randomized, placebo-controlled, sequential group first-in-human study of the TRPV1 antagonist, JNJ-38893777, in healthy Men. Clin Drug Investig.

[60] Honore P, Chandran P, Hernandez G, Gauvin DM, Mikusa JP, Zhong C, Joshi SK, Ghilardi JR, Sevcik MA, Fryer RM, Segreti JA, Banfor PN, Marsh K, Neelands T, Bayburt E, Daanen JF, Gomtsyan A, Lee C-H, Kort ME, Reilly RM, Surowy CS, Kym PR, Mantyh PW, Sullivan JP, Jarvis MF, Faltynek CR (2009). Repeated dosing of ABT-102, a potent and selective TRPV1 antagonist, enhances TRPV1-mediated analgesic activity in rodents, but attenuates antagonist-induced hyperthermia. Pain.

